# Topics of Public Interest

**Published:** 1907-11

**Authors:** 


### TOPICS OF PUBLIC INTEREST.

### Typhoid from Oysters

**Shell Fish in Local Waters Contaminated by Sewage.**

(*New York Tribune*, October 8, 1907)

THE danger of typhoid from oysters and clams has been pointed out many times, but a new note of alarm was sounded yesterday by Dr. Daniel Lewis, president of the Metropolitan Sewage Commission, and Dr. Daniel D. Jackson, bacteriologist of the New York water board. They both declared that many of the oysters supplied to the New York market come from neighboring waters that are literally nothing more than a strong solution of sewage.

Although it has been known that New York Bay is badly contaminated, few persons suppose that these waters were used to breed oysters which are sold under names that suggest they come from far away and clean localities. The pollution committee of the Merchants' Association has estimated that nearly 1,000,000,000 gallons of sewage is discharged into the bay every day, and that of the 2,000 tons of sewage thus deposited fully 700 tons remain in suspension. The theory that the outgoing tide carries away this filth has been overturned by investigation. and it is now accepted as a fact that 79 per cent. of the water which goes out with the tide comes back again. And yet, in the face of this, the map prepared by the New York Pollution Committee shows that there is a bed of oysters near the Statue of Liberty, and that here 50,000 bushels of oysters are gathered annually.

In discussing this question yesterday Dr. Lewis said:

There isn't a square foot of the waters of the harbor that isn't polluted by sewage. Newark Bay is in a disgraceful condition, and the waters of the bay on the south shore of Staten Island, where most of the oysters and clams are gathered, is nothing more or less than a whirlpool of sewage.

I would no more think of eating an oyster or clam taken from these waters than I would so much deadly poison. When president of the New York Bay Pollution Commission I made an extensive examination of the subject, the results of which are embodied in the first report of that commission. Of the thousands of oysters and clams analysed there wasn't one that did not show traces of sewage. The matter is a crying outrage, yet nothing has been done by the authorities of New York and New Jersey to stop it.

It has been proved by experts that only a small percentage of the 1,000,000,000 gallons of sewage which daily finds its way into New York Harbor ever gets into the sea. Just as the ebb tide gets past the Narrows it is met by the flood tide, and the sewage is swirled back into the bay again, and in these filth laden waters more than 600,000,000 oysters and clams are annually harvested, mainly for the New York markets.

One particular outrage that I would like to have the attention of the public called to is the fact that countless thousands of oysters are "fattened" in the Raritan and Elizabeth rivers, which are little short of running sewers. It is in these waters, as I understand it, that immense quantities of oysters of various fancy names are "cultivated" for the fashionable New York hotels and restaurants.

As is generally known, the Shrewsbury River is vilely polluted. No one now hears of the formerly celebrated Shrewsbury oyster. They are simply called Lynnhavens instead. And so on through the chapter. I am glad to see that the New Jersey State Sewerage Commission has just served notice on the city of New Brunswick to show cause why it should not be ordered to cease polluting the Raritan River. The pollution of the Elizabeth River is also before the courts.

The fall of the year always shows a marked increase in the number of typhoid fever cases. For instance, during the week ended August 13, 68 cases were reported with 13 deaths. During the week ended August 24, 53 cases were reported with 17 deaths. The record for the week ended September 21 shows that 181 cases were reported, with a death list of 27. In midsummer, when very few oysters are eaten, the death-rate from typhoid fever sinks almost to the zero mark.

The waters of the harbor and vicinity have now become so polluted by sewage and factory waste that there is scarcely an edible fish left. Think, then, of its effect upon shell fish. The time is rapidly approaching when New York will have to devise some method of disposing of the sewage other than discharging it into the bay, for if continued for many years longer the health of the whole community will be seriously threatened.

Dr. Jackson, in talking on the same subject, said that he believed fully 5 per cent. of the cases of typhoid in this city were due to the eating of infected shell food. "Virtually none of the enormous quantities of filth thrown into our harbor by the many sewers," he said, " is disposed of outside the harbor. I believe that a stop should be put to the taking of oysters from these waters. It is a constant risk to health and life. Of course, in time some way will be hit upon to purify our waters, or with the increase of population in the Hudson Valley the river and harbor will become a cesspool. As matters stand I would advise everybody to be sure of the source of their sea food. I have examined oysters from Gravesend Bay and the shores of Staten Island and found in them filth that might cause any number of intestinal diseases."

